# Nutrition as Metabolic Treatment for Anxiety

**DOI:** 10.3389/fpsyt.2021.598119

**Published:** 2021-02-12

**Authors:** Nicholas G. Norwitz, Uma Naidoo

**Affiliations:** ^1^Department of Physiology, Anatomy and Genetics, University of Oxford, Oxford, United Kingdom; ^2^Harvard Medical School, Boston, MA, United States; ^3^Department of Nutrition and Lifestyle Psychiatry, Massachusetts General Hospital, Boston, MA, United States

**Keywords:** anxiety, inflammation, microbiome, nutrition, mental illness

## Abstract

Despite the overwhelming prevalence of anxiety disorders in modern society, medications and psychotherapy often fail to achieve complete symptom resolution. A complementary approach to medicating symptoms is to address the underlying metabolic pathologies associated with mental illnesses and anxiety. This may be achieved through nutritional interventions. In this perspectives piece, we highlight the roles of the microbiome and inflammation as influencers of anxiety. We further discuss the evidence base for six specific nutritional interventions: avoiding artificial sweeteners and gluten, including omega-3 fatty acids and turmeric in the diet, supplementation with vitamin D, and ketogenic diets. We attempt to integrate insights from the nutrition science-literature in order to highlight some practices that practitioners may consider when treating individual patients. Notably, this piece is not meant to serve as a comprehensive review of the literature, but rather argue our perspective that nutritional interventions should be more widely considered among clinical psychiatrists. Nutritional psychiatry is in its infancy and more research is needed in this burgeoning low-risk and potentially high-yield field.

## Introduction

Anxiety disorders are the most common type of psychiatric condition in the United States, with one-third of individuals suffering from some form of anxiety during their lifetime (1). Standard of care medications and psychotherapy are only successful in treating about half of patients, and only one-quarter experience complete symptomatic resolution (2).

While medications and behavioral therapies certainly have their place as part of a multifaceted approach to treat anxiety, the relatively high failure rate of such approaches is consistent with the broader failure of drug treatments for most neurological conditions. For example, antidepressants are efficacious in only about one-third of clinical cases (3) and there are no established disease-modifying medications for major neurodegenerative conditions like Parkinson's disease or Alzheimer's disease. With respect to the latter, the drug discovery failure rate for mere symptomatic management is 99.6% (4). It is therefore feasible, if not probable, that we are approaching neurological conditions with the wrong paradigm. As neurological conditions and mental illnesses (5) are characterized by a subset of fundamental metabolic disturbances [such as oxidative stress (6), insulin resistance (7), inflammation (8), and microbiome dysbiosis (9)] to which lifestyle factors are a contributor, it would makes sense that mental illnesses deserve complementary lifestyle approaches. In effect, lifestyle interventions for mental illness are a form of metabolic medicine complementary to metabolic disease (5). Nutrition is one such metabolic medicine.

Herein, we discuss the pathological correlates of anxiety disorders specifically, emphasizing the possible roles of microbiome dysbiosis and inflammation. We chose to structure this perspective piece as follows: First, we discuss microbiome dysbiosis and inflammation, pathologies that are particularly relevant to anxiety disorders in order to establish anxiety as a metabolic disease. Second, we discuss six nutritional strategies for which there is emerging evidence of their efficacy in anxiety. These are elimination of (i) artificial sweeteners and (ii) gluten, inclusion (iii) omega-3 fatty acids and (iv) turmeric (curcumin), maintaining adequate levels of (v) vitamin D, and (vi) and ketogenic diets. Within each section, we build up the evidence hierarchy from a mechanistic metabolic perspective, to animal models, to human studies. The purpose of this piece is not to delve into all the mechanisms of interventions (for which there is currently limited data), but demonstrate that anxiety is a metabolic disease and that nutritional therapy can be efficacious in its treatment.

## Microbiome

The gut contains ~40 trillion microorganisms and is the largest endocrine organ in the body. By communicating to the brain via the Vagus nerves, regulating hormones, and influencing inflammation, the gut can impact mental health (10). More specifically, the compositions of individuals' gut microbial ecosystems can regulate mental status and anxiety (11). It is, therefore, unsurprising that microbiome dysbiosis is associated with anxiety (9).

As a comprehensive description of the mechanisms by which the microbiome and gut-brain axis influence the neuroanatomy and neurochemistry of anxiety is beyond the scope of this piece, we will emphasize the role of the amygdala, short chain fatty acids (SCFAs), and gut peptides as examples [Please see the following references as starting points for further reading on the Vagus nerve (12) or microbiome influence of cytokine production (13)].

The amygdala is a structure in the brain largely responsible for the threat response that is hyperactive in anxiety disorders (14). Interestingly, germ-free mice exhibit larger and more active amygdalae (15, 16). Furthermore, fecal transplantation, or the introduction *Bifidobacterium infantis*, has been shown to correct excessive stress response in such germ-free mice (17), implicating the microbiome in amygdala dysfunction.

The amygdala has receptors for gut peptides, including neuropeptide Y (NPY), pancreatic polypeptide (PP), and glucagon-like peptide 1 (GLP-1) (11, 18). Addressing each of these, there is evidence that the NPY system affects anxiety (19, 20); the PP Y_4_ receptor has been shown to modulate anxiety in rodents (21); and multiple GLP-1 receptor agonists have been used to address anxiety in animal models (22, 23). The release of each of these gut peptides is regulated by SCFAs produced by certain gut bacteria, which act through the G-protein coupled receptors, free fatty acid receptors 2 (FFAR2) and FFAR3 (11). Notably, populations of SCFA producing species tend to be reduced in individuals with anxiety (9).

In review, food influences the microbiome (24) and microbe-derived SCFAs bind to receptors on enteroendocrine cells to regulate the secretion of gut peptides, which themselves bind to receptors on the amygdala to influence the stress response and anxiety. This is just one cascade by which diet can influence the brain. SCFAs from gut microbes can also act through immune, inflammatory, and other endocrine mechanisms (25, 26), and lipopolysaccharide (LPS) from gram-negative bacteria can induce anxiety when leaked into circulation through a compromised gut barrier (27, 28). The mechanisms are many, but the point is simple: diet and nutrition influence anxiety by modulating the microbiome.

It is also to be emphasized that the microbiome-brain axis is a bidirectional relationship. Negative emotions can shift the microbial ecosystem by the release of stress hormones sympathetic neurotransmitters (29). Therefore, even if the current state of science does not enable precision medicine aimed at the microbiome, it is still important to consider the role that positive feedback loops between the gut and brain may be playing in anxiety disorders.

## Inflammation

Chronic inflammation is a feature of almost all neurological and neurodegenerative disorders, including anxiety (30). Individuals suffering from anxiety and anxiety-related disorders, like panic disorder (31), generalized anxiety disorder (32), and post-traumatic stress disorders (PTSD), exhibit elevated levels of inflammatory markers in their circulation and cerebral spinal fluid (33). These include C-reactive protein (CRP), IL-1β, IL-6, and TNFα (31, 34–38). These cytokines contribute to neurotransmitter imbalances in the brain (including, serotonin, dopamine, glutamate/GABA) and can pathologically increase amygdala responsivity (30).

Suggestions of causality exist in the literature and, because the existing literature is most highly focused on PTSD, we too will focus on PTSD as a case in point of potential causality. As examples, polymorphisms in *CRP* predict increased likelihood of being diagnosed with PTSD, and predict worse symptoms if diagnosed (36); a study on immune cells taken from patients with anxiety showed increased reactivity and secretion of the cytokines, IL-17 and TNFα (39); and, administration of LPS to 39 healthy subjects doubled amygdala activity, as measured by fMRI, in response to socially threatening images (40). Admittedly, the state of research on the mechanisms of inflammation-induced anxiety is in its infancy. Nevertheless, it is probable that inflammation contributes to anxiety in at least some, and possibly a majority, of patients.

The menu of “inflammatory foods” is extensive, but generally includes foods associated with the Standard American Diet (SAD). Holistically speaking, the two most metabolically challenging components of SAD are refined sugars and processed vegetable oils, both of which can contribute to inflammation through myriad mechanisms (41–43). To mention a few as illustrative points, refined sugars, and in particular high fructose corn syrup, contribute to *de novo* lipogenesis of pro-inflammatory visceral fat (43), and fructose now composes 10% of caloric intake in the United States (42). Sugar also attaches to molecules throughout the body to generate inflammatory advanced glycation end products (AGEs). It has even been demonstrated that sugar can increase the production of AGEs in the brain and that these AGEs increase neuroinflammation and contribute to metabolic diseases (41).

Processed vegetable oils, such as corn oil and soybean oil, that contain high levels of the omega-6 fatty acids, linoleic acid, are likewise inflammatory. Having been stripped of the antioxidants that protect omega-6 fats in whole foods, the linoleic acid in processed vegetable oils incorporates into cells and tissue throughout the body, gets oxidized, and can initiate a vicious cycle of oxidation, insulin resistance, and inflammation that perpetuates metabolic and inflammatory diseases from the gut to the brain (7, 44–46). Increased consumption of linoleic acid-containing vegetable oils has even been proposed as a driver of cardiovascular disease (47), an inflammatory disease and comorbidity of anxiety disorders (46, 48, 49). Elimination of refined sugars and processed vegetable oils from the diet, and their replacement with whole foods, is foundational for good physical, cognitive, and mental health. However, more specific dietary and nutritional interventions have been explored in the context of anxiety, and it is to these which we turn.

## Nutritional Strategies

### Artificial Sweeteners

Administration of artificial sweeteners to animals has been shown to precipitate anxiety (50). The anxiolytic effects of sweeteners are likely mediated by their adverse impacts on the microbiome and inflammation. Negative effects of certain sweeteners on systemic metabolism have been shown to be causal in animal models and humans, although the precise pathways are unknown (51, 52). Other mechanisms exist as well. For example, aspartame given to rats increased the levels of stress hormones in the animals' amygdalae (53). Aspartame can also block the transport of dopamine and serotonin precursors into the brain and can increase the levels of excitatory neurotransmitters, shifting brain chemistry toward an anxiety prone state (54).

In humans, artificial sweeteners have been associated with neuropsychiatric problems, including anxiety (55). Further, it has been proposed that individuals suffering from mental disorders may be particularly susceptible to the adverse effects of artificial sweeteners. For example, a randomized, placebo-controlled, crossover study designed to assess the impact of aspartame on mood was prematurely terminated because of the severity of reactions in patients with a history of depression (56), which is highly comorbid with anxiety (57).

Unfortunately, the literature is currently limited to the investigation of only a narrow range of sweeteners (and predominantly the sweetener, aspartame, found under the trade names *Equal* and *NutraSweet*, and in popular low-fat snacks and drinks, like Diet Coke). Future human studies will hopefully reveal associations between specific sweeteners and specific neurological disorders so that nutritional psychiatrists can provide more specific recommendations.

For patients unwilling to give up sweeteners, stevia (a natural non-caloric, non-insulinogenic sweetener) and erythritol [a non-insulinogenic sugar alcohol that gets absorbed in the small intestine and is not fermented by gut bacteria (58)] may be reasonable alternatives to recommend to patients in a practical clinical setting because they are presumed to have minimal negative impact on insulin sensitivity and the microbiome and are, therefore, less likely to cause metabolic dysfunction. However, as absence of evidence does not equate to evidence of absence, the most conservative approach is still the complete elimination of sugar and sweeteners.

### Gluten

Gluten can induce inflammation by causing “leaky gut.” Gluten proteins increase zonulin expression, which increases gut permeability (59, 60). Thereafter, immune stimulating compounds, like LPS, leak from the gut into the bloodstream, leading to inflammation.

Zonulin protein is overexpressed in celiac disease, a condition that itself is associated with social phobias, panic disorder, and other forms of anxiety (61–63). Generalizing beyond celiac disease, zonulin has been linked as a biomarker of mental illnesses such as autism, attention deficient hyperactivity disorder, and schizophrenia (64). Even in anxiety patients with no reported history of gastrointestinal disturbances, zonulin and LPS are found at elevated levels in the blood relative to non-anxious control subjects (65). This is consistent with the hypothesis that gluten can cause “leaky gut” to precipitate inflammation and anxiety and suggests patients with anxiety may be particularly sensitive to gluten.

At this time, a gluten-free diet has been shown to decrease anxiety only in celiac patients (66). Nevertheless, we feel it is reasonable to include a gluten-free diet in the arsenal of metabolic treatments for anxiety, given the mechanistic link to “leaky gut” and associations between zonulin and mental illness and zonulin levels and anxiety.

### Omega-3s

Omega-3 fatty acids, particularly the long-chain omega-3s, eicosapentaenoic acid (EPA) and docosahexaenoic acid (DHA), are potent anti-inflammatory signaling molecules that support the microbiome (67, 68) and are important in cognition and mental health (69, 70). Direct evidence that omega-3s themselves are healthful, in addition to their whole food sources, comes from the comparison of genetically engineered mice that can biosynthesize omega-3 and/or omega-6 fats. On identical diets, mice that biosynthesize omega-3s and have lower omega-6/omega-3 ratios and exhibit healthier microbiomes, less inflammation, and less chronic disease (71). In preclinical studies on rats suffering from inflammation-induced anxiety, omega-3-rich diets have been shown to normalize dopamine levels (72) and reduce anxiety-like behaviors (73). And, in mice, omega-3s have been shown to improve serotonergic neurotransmission and increase levels of brain-derived neurotrophic factor (BDNF) (74). Thus, while the mechanisms by which omega-3s assist in addressing the metabolic foundations of anxiety are manifold, they likely include improving microbiome balance, decreasing inflammation, and balancing neurochemistry.

Turning to humans, Green et al. demonstrated that, in patients with social anxiety disorder, erythrocyte EPA and DHA levels are reduced 18–34%. Moreover, an inverse correlation exists between levels of these omega-3s and severity of anxiety (75). Similar observations have been made by others (76), and these associations are backed by interventional trials.

A randomized, double-blinded, placebo-controlled trial on 68 medical students showed that 12 weeks of omega-3 supplementation lowered anxiety by 20%. This study also revealed that lower omega-6/omega-3 ratios predicted lower levels of inflammatory markers and anxiety (77). Lastly, a meta-analysis of nineteen clinical trials, including 2,240 participants across eleven countries, concluded that omega-3 treatment is effective in reducing anxiety (78).

The aforementioned meta-analysis also highlights the fact that dose and omega-3s type are important to consider. Studies that used doses lower than 2 grams per day tended not to be effective in treating anxiety. Furthermore, subgroups analyses found that supplements with lower proportions of DHA were less effective in reducing anxiety, with supplements containing more than 60% EPA having no significant effect (78).

Practically speaking, on the topic of omega-3 types and sources, plant sources of omega-3 (such as flax seeds and chia seeds) contain primarily alpha linolenic acid (ALA), a shorter chain omega-3 that is converted in to the more bioactive EPA and DHA only at very low levels, on the order of 5% conversion (79–81). Fatty fish, such as mackerel, sardines, and Alaskan sockeye salmon are far richer in EPA and DHA. Salmon, in particular, includes the antioxidant, astaxanthin, which not only gives salmon their pink-red color but also protects omega-3s from oxidation and itself has neuroprotective properties (82). It is also worth mentioning that there is diversity among DHA forms. Specifically, lysophosphatidylcholine-conjugated DHA, found at its highest levels in fish roe and krill oil, has privileged transport to the brain via the major facilitatory superfamily domain-containing protein (MSFD2A) transporter, a transmembrane protein that exists within endothelial cells at the blood-brain barrier. Whereas, free DHA bound in the blood crosses into the brain via passive diffusion, the MSFD2A transporter actively shuttles lysophosphatidylcholine-conjugated DHA into the brain using energy derived from the sodium electrochemical gradient (83). This active transport mechanisms may be particularly beneficial in inflamed brains in which the blood-brain barrier is compromised. Therefore, when recommending omega-3 sources to patients, fish roe and krill oil may be the best options, followed by salmon and other fatty fish.

Thus, there is mechanistic rationale, animal and human data supporting the emphasis of dietary omega-3 for the treatment of anxiety.

### Turmeric (Curcumin)

Turmeric is probably the most heavily studied spices for brain health. Its active component, curcumin, has been explored as a treatment for Alzheimer's disease, Parkinson's disease, depression, comorbidities of anxiety, and anxiety itself (84, 85). Curcumin's mechanisms of action are many and include improving the gut microbial ecosystem (86), decreasing inflammation by inhibiting NFκB and the NLRP3 inflammasome (87–90), altering dopamine, serotonin, and cortisol levels (91), and regulating microRNAs and histone deacetylases (HDACs) (92).

Preclinical trials of curcumin for anxiety in rodent models add to the promise of curcumin as an anti-anxietolytic. In rats treated with a food preservative to induce anxiety, curcumin treatment completed rescued anxiety-like behaviors (93). Similar findings have been reported in other animal models of anxiety (94, 95). In these and other studies, curcumin significantly reduced anxiety-like behaviors concomitant with complementary improvements in neurotransmitter and hormone levels (91, 94, 95).

Multiple randomized, double-blinded, placebo-controlled trials have shown that curcumin supplementation can reduce anxiety in human patients. In patients with diabetes, 8 weeks of curcumin supplementation decreased anxiety (96). A crossover trial on 30 obese individuals likewise found that curcumin supplementation for 30 days reduced anxiety scores (97). And, a meta-analysis of five studies reported an overall significant effect of curcumin on anxiety with a large effect size [Hedge's *g* = −2.62 (84)].

Admittedly, there are limitations to the curcumin literature. Some have challenged that the health benefits of turmeric and its active components are over sensationalized. Specifically, Nelson et al. performed a careful analysis of the medical chemistry of curcumin and make a compelling case that the positive results in model systems may be confounded by curcumin's chemical instability and potential for interfering with assay readouts. Furthermore, they point out that there is a great degree of variability among studies with respect to supplement purity and formulations, which confound the reproducibility of studies (98). For example, curcuminoids are fat-soluble and exhibit <1% bioavailability when administered alone or an aqueous solution (99). For this reason, curcuminoids should be consumed with fats, and lipid-based delivery systems have been and are being developed for the administration of curcumin, including liposomes and nanoparticles (100). Indeed, the two randomized controlled trials referenced in the previous paragraph that reported positive findings for curcumin on anxiety each employed techniques to increase the bioavailability of curcumin, including nano-curcumin (96) and co-administration of bioperine (97) (also known as piperine), which enhances curcumin absorption 20-fold (101). Thus, future research resources should be devoted to the study of these more bioavailable forms of curcumin, and also to the impact of curcumin on the human microbiome, as this does not require systemic absorption.

Turmeric also relates to the previous section on omega-3s. Recall that humans are inefficient at converting ALA into EPA and DHA. Curcumin can increase ALA to DHA conversion by increasing levels of the DHA synthesis enzymes (102). This not only increases DHA in the brain but has direct functional implications on anxiety. Rodents treated with a combination of ALA and curcumin exhibit decreased anxiety (102).

### Vitamin D

As most modern humans spend most of their time inside, fully clothed, or simply living at high latitudes, endogenous vitamin D production is often inadequate. It is also difficult to get enough vitamin D from the diet. Even using the most favorable numbers for vitamin D content of milk, one would need to consume five gallons of whole milk daily to meet the recommended 600 IU (103), ignoring that many practitioners believe higher doses may be optimal. On a population level, vitamin D insufficiency (<30 ng/mL) has been estimated at 77% in the United States (104), making low vitamin D levels a hormonal epidemic.

In the brain, vitamin D regulates calcium homeostasis and ion channels (105, 106), neurotransmitter levels, including dopamine and serotonin (107–110), and the secretion of nerve growth factor and BDNF (111, 112). The benefits of vitamin D are also likely mediated by its role in shaping the microbiome and reducing inflammation (113–117).

Lower levels of vitamin D are associated with multiple mental disorders, including schizophrenia (118), depression (119), and anxiety (120–122). One such association study found that vitamin D levels in patients with a wide range of anxiety disorders were <60% those of healthy controls (120).

In interventional studies, vitamin D supplementation to those with vitamin D deficiency has been effective in addressing anxiety. In a study of 30 anxiety patients, once weekly vitamin D supplementation at 50,000 IU for 3 months significantly improved symptoms (123). A similar study in 51 women with type II diabetes also showed that 50,000 IU vitamin D fortnightly decreased inflammation and reduced symptoms of anxiety over 4 months (124).

It may be that vitamin D supplementation for anxiety is only effective in those with vitamin D insufficiency, with one association study finding elevated anxiety only on those with extreme vitamin D deficiency (<10 ng/mL) (125). Nonetheless, vitamin D insufficiency persists amongst Americans, sufficient vitamin D intake is difficult to obtain through diet or sun exposure, when living at higher latitudes, and adequate vitamin D levels are important to overall health. Therefore, vitamin D supplementation should be considered for patients with anxiety as most will be vitamin D insufficient and the collateral effects on patient health are likely to be positive.

### Ketogenic Diets

Ketogenic diets—high-fat, low-carbohydrate diets that induce the body to produce ketones, a fuel source for the brain—are gaining traction as a metabolic treatment for a wide range of chronic metabolic diseases (126–131). Ketogenic diets have been used for a century to treat drug-resistant pediatric epilepsy, are still widely used for epilepsy (132, 133), and are gaining in popularity for the treatment of neurodegenerative conditions, such as Parkinson's disease (131, 134, 135) and Alzheimer's disease (128, 130, 136). For reviews specifically on the topic of ketogenic diets for neurological diseases and mental illnesses, we recommend the following recent reviews, both published this year, which cover the literature supporting the therapeutic implementation of ketogenic diets for a wide range of conditions including attention deficit hyperactivity disorder (ADHD), bipolar disorder, schizophrenia, autism spectrum disorder, major depressive disorder, and binge eating disorder (5, 137).

Ketogenic diets help to address many of the biopathological foundations of chronic neurological diseases and mental illnesses, including glucose hypometabolism, neurotransmitter imbalances, oxidative stress, and inflammation (5). Ketones produced by the liver during carbohydrate restriction are not only a more efficient fuel substrate for the brain, but are also signaling molecules that bind their own G-protein coupled receptors, inhibit HDACs, directly modify histones, shift the gut microbiome and improve gut barrier function, and reduce oxidative stress and inflammation (134, 138–142).

Preclinical models offer promise. Rats orally administered exogenous ketone supplements to achieve ketone levels comparable to those achieved by patients on ketogenic diets (~0.8 mmol/L) exhibited significant decreases in anxiety (143). Another rat study found that ketosis induced anti-anxiolytic changes in brain metabolism in association with a reduction of anxiety-like behaviors (144). There is also evidence that intermittent fasting, an intervention that induces ketosis, induces neurological adaptations overtime (including an upregulation of the mitochondrial sirtuin, SIRT3, and GABAergic activity) that and neuroprotective and reduce anxiety (145). No clinical trials assessing the efficacy of ketogenic diets for anxiety have yet been conducted. We include ketogenic diets as a potentially promising future option and to provide clinicians with a perspective on the emerging research on nutrition and mental health.

### Other Strategies

Additional nutritional strategies hold potential for the treatment of anxiety disorders, including caffeine reduction (146, 147), prebiotics (148, 149), and probiotics (150) to support the microbiome, and supplementation with or magnesium (151, 152) or tryptophan (153) to potentially increase serotonin synthesis.

## Conclusion

**Figure 1:**
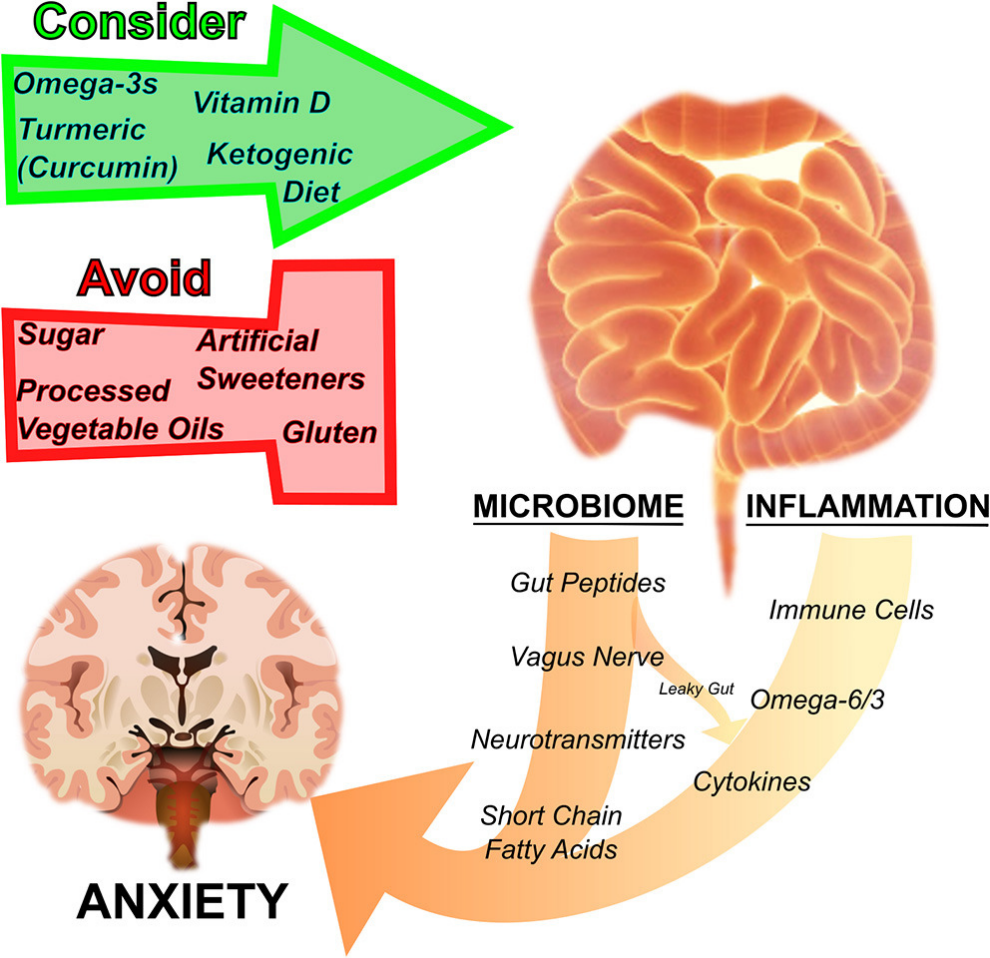
Nutrition regulates anxiety disorders by influencing the microbiome and inflammation. The gut microbiome and inflammation are interrelated and influence anxiety. By acting on the gut microbial ecosystem, regulating inflammation, as well as through other pathways mentioned in the text, particular nutritional strategies have been suggested to either harm or help disorders of anxiety. Sugar, processed vegetable oils rich in inflammatory omega-6 fatty acids, artificial sweeteners, and gluten have a negative effect on anxiety, whereas omega-3 fatty acids, turmeric (curcumin), vitamin D, and ketogenic diets are thought to have a therapeutic effect.

Herein, we provide biological rationales and translational evidence that nutritional strategies aimed at addressing disturbances in metabolism and brain function can protect against anxiety disorders (Figure 1). Anxiety and other mental illnesses are metabolic diseases as much as they are psychological. And, in our opinions, metabolic diseases deserve metabolic medicine. Nutrition is one form of metabolic medicine, and one which patients and clinicians interact with every day. It is important to leverage this metabolic tool to better offer persons suffering with anxiety a full spectrum of relief.

However, the clinical challenge of bioindividuality persists. Different patients are afflicted with different deficiencies and comorbidities. We each carry genetic polymorphisms and have distinct microbiomes. Therefore, future research should be focused on determining the mechanisms by which various interventions operate such that the medical community can turn nutritional psychiatry from a shotgun approach into precision personalized medicine.

In closing, we pose the question, “if patients needs to eat everyday anyway, why not turn a gustatory pleasure into an experimental one as well?”

## Data Availability Statement

The original contributions presented in the study are included in the article/supplementary material, further inquiries can be directed to the corresponding author/s.

## Author Contributions

Both authors contributed to the work and approved it for publication.

## Conflict of Interest

NN and UN each declare that they each stand to receive royalties from their respective books, “The New Mediterranean Diet Cookbook” and “This is Your Brain of Food”.

## Abbreviations

AGEs: advanced glycation end products
ALA: alpha-linolenic acid
BDNF: brain-derived neurotrophic factor
CRP: C-reactive protein
DHA: docosahexaenoic acid
EPA: eicosapentaenoic acid
FFARs: free fatty acid receptors
GABA: gamma-aminobutyric acid
GLP-1: glucagon-like peptide 1
HDACs: histone deacetylases
IBDs: inflammatory bowel diseases
IBS: irritable bowel syndrome
IL: interleukin
LPS: lipopolysaccharide
MSFD2A: major facilitator superfamily domain-containing protein 2 A
NFκB: nuclear factor κ-light-chain enhancer of activated B cells
NLRP3: NOD-LRR-and pyrin domain-containing protein 3
NPY: neuropeptide Y
PP: pancreatic polypeptide
PTSD: post-traumatic stress disorder
SAD: Standard American Diet
SCFAs: short chain fatty acids
TNFα: tumor necrosis factor α.
